# Non-Invasive Ultrasound Therapy for Severe Aortic Stenosis: Early Effects on the Valve, Ventricle, and Cardiac Biomarkers (A Case Series)

**DOI:** 10.3390/jcm13164607

**Published:** 2024-08-07

**Authors:** Danijela Trifunović-Zamaklar, Radmila Karan, Nataša Kovačević-Kostić, Duško Terzić, Vladimir Milićević, Olga Petrović, Ivana Canić, Mathieu Pernot, Mickael Tanter, Louise Z. Wang, Guillaume Goudot, Miloš Velinović, Emmanuel Messas

**Affiliations:** 1 Faculty of Medicine, University of Belgrade, 11000 Belgrade, Serbia; 2 Clinic for Cardiology, University Clinical Center of Serbia, 11000 Belgrade, Serbia; 3 Department for Anesthesia and Intensive Care at Clinic for Cardiac Surgery, Centre for Anesthesiology and Reanimatology, University Clinical Centre of Serbia, Pasterova 2, 11000 Belgrade, Serbia; 4 Cardiosurgery Department, University Clinical Center of Serbia, 11000 Belgrade, Serbia; 5 Centre for Medical Biochemistry, University Clinical Centre of Serbia, 11000 Belgrade, Serbia; 6 Physic for Medicine, Inserm, ESPCI, CRNS, PSL Research University, 75015 Paris, France; 7 Cardiovascular Department, Hôpital Européen Georges-Pompidou, Université Paris-Cité, 75015 Paris, France; 8 RHU STOP-AS Research Consortium, 76031 Rouen, France; 9 Paris Cardiovascular Research Center, INSERM UMR U970, 75015 Paris, France

**Keywords:** aortic valve stenosis, non-invasive ultrasound therapy, aortic valve hemodynamics, global longitudinal strain, myocardial work index

## Abstract

**Background**: Transcatheter aortic valve replacement (TAVR) was developed for inoperable patients with severe aortic stenosis. However, despite TAVR advancements, some patients remain untreated due to complex comorbidities, necessitating less-invasive approaches. Non-invasive ultrasound therapy (NIUT), a new treatment modality, has the potential to address this treatment gap, delivering short ultrasound pulses that create cavitation bubble clouds, aimed at softening embedded calcification in stiffened valve tissue. **Methods**: In the prospective Valvosoft^®^ Serbian first-in-human study, we assessed the safety and efficacy of NIUT and its impact on aortic valve hemodynamics, on the left ventricle, and on systemic inflammation in patients with severe symptomatic aortic stenosis not eligible for TAVR or surgery. **Results**: Ten patients were included. Significant improvements were observed in hemodynamic parameters from baseline to one month, including a 39% increase in the aortic valve area (from 0.5 cm^2^ to 0.7 cm^2^, *p* = 0.001) and a 23% decrease in the mean transvalvular gradient (from 54 mmHg to 38 mmHg, *p* = 0.01). Additionally, left ventricular global longitudinal strain significantly rose, while global wasted work significantly declined at one month. A dose–response relationship was observed between treatment parameters (peak acoustic power, intensity spatial-peak pulse-average, and mean acoustic energy) and hemodynamic outcomes. NIUT was safely applied, with no clinically relevant changes in high-sensitivity troponin T or C-reactive protein and with a numerical, but not statistically significant, reduction in brain natriuretic peptide (from 471 pg/mL at baseline to 251 pg/mL at one month). **Conclusions**: This first-in-human study demonstrates that NIUT is safe and confers statistically significant hemodynamic benefits both on the valve and ventricle.

## 1. Introduction

The prevalence of aortic stenosis is rising rapidly due to an aging population [1]. Transcatheter aortic valve replacement (TAVR) was developed to provide a treatment for patients that would have otherwise been left untreated, offering a less-invasive approach compared to surgical aortic valve replacement [2]. Meanwhile, TAVR has an established role in treating patients with aortic stenosis [1,3].

However, even in the TAVR era, a substantial number of patients remain untreated. In a meta-analysis and modelling study, the annual incidence of severe aortic stenosis was estimated at 4.4‰ in patients ≥65 years, with approximately two-thirds of these having severe symptomatic aortic stenosis. A total of 58% of them receive surgical aortic valve replacement, while from the remaining 42%, only 61.7% receive TAVR [4]. Thus, around 16% of patients with severe symptomatic aortic stenosis are left untreated.

Accordingly, the next frontier in treating aortic stenosis is to offer patients a less-invasive strategy to block or impede the course of the pathology [5,6]. Pulsed cavitational ultrasound therapy, creating shockwaves, has been established as a safe therapy in coronary and peripheral calcified arteries and now targets aortic valve stenosis [6,7]. The therapy does not remove the calcium and, thus, does not risk resulting in thrombotic events but rather aims to restore the softness and pliability of the tissue [6].

One concept is the invasive use of shockwaves via percutaneous access. The feasibility of this concept has so far been reported in animal and ex vivo studies and in case reports [6,7,8,9]. Another concept is the use of non-invasive ultrasound therapy (NIUT) that uses the same principle but is non-invasive.

The novel Valvosoft^®^ device (Cardiawave^®^, Levallois-Perret, France) uses NIUT applied to the patient’s chest. It has been designed to deliver focused ultrasound pulses of high acoustic pressure amplitudes to produce cavitation bubble clouds that aim to act on embedded calcification, softening the stiffened calcified aortic valve. The device has been successfully tested in animals confirming the non-invasiveness of the device through histopathological assessments [5,10], and its initial safety and efficacy have been confirmed in first-in-human studies [11,12]. 

The outcomes of the Valvosoft^®^ Serbian first-in-human study were summarized in a research letter [11]. However, holistic analysis of the biological effects of (any) innovative therapy for aortic stenosis should be carried out in accordance with the contemporary paradigm that aortic stenosis is a disease of both the valve and the ventricle [13], and it should also elucidate the safety of the therapy, including its effects on the heart (myocardium) and other organs (brain), as well as systemic effects (on hemostasis and inflammation). 

In this paper, we aimed to interrogate the early effects of NIUT on three groups of parameters: (a) aortic valve hemodynamics (such as peak systolic velocity, Vmax; mean transvalvular gradient, and aortic valve area, AVA; all assessed by transthoracic Doppler echocardiography) and valve shear stress (estimated by the changes in von Willebrand factor, vWF); (b) left ventricular systolic function (ejection fraction), left ventricular wall stress (brain natriuretic peptide, BNP), and GLS and myocardial work; and (c) potential myocardial injury induced by the treatment and systemic inflammatory response to it (measured using high-sensitivity Troponin T, hs-TnT; and C-reactive protein, CRP). We assessed these predefined parameters at three time points: before treatment, at hospital discharge one day after NIUT, and at one month after NIUT.

## 2. Materials and Methods

### 2.1. Study Design

The Valvosoft^®^ Serbian first-in-human study is a prospective, single-arm, single-center study conducted in Serbia between December 2019 and May 2022. The TAVR program was not fully developed in Serbia at that time. Follow-up was planned at 1, 3, 6, 12, and 24 months.

Patients were eligible if they had severe symptomatic aortic stenosis and were not eligible for TAVR/surgical aortic valve replacement or refused such treatment. The full list of inclusion and exclusion criteria is provided in Supplemental Table S1. A multidisciplinary heart team decided on the eligibility of the patient.

The study was performed in accordance with the Declaration of Helsinki and ISO14155 (Clinical investigation of medical devices for human subjects—Good clinical practice) in its current version. All procedures have been performed in compliance with relevant laws and institutional guidelines and have been approved by the appropriate institutional ethic committee (ref #850/9). All patients provided their written informed consent prior to any study procedure. Data monitoring was performed by an independent clinical research organization (MD Clinicals, Saint-Sulpice, Switzerland), a clinical event committee adjudicated all adverse events, and an independent data and safety monitoring board monitored the safety and performance of the device.

### 2.2. Study Device and Procedure

**Figure 1:**
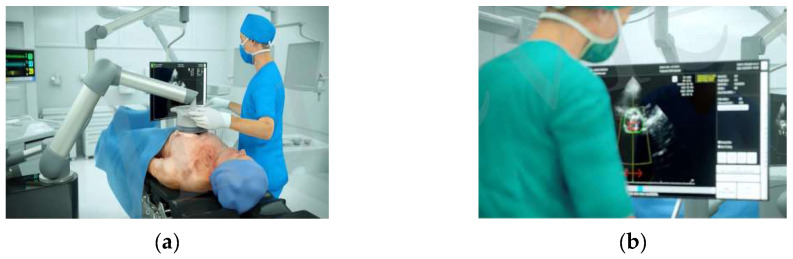
Valvosoft device using non-invasive ultrasound therapy. During treatment, the applicator, which contains an imaging ultrasound and an independent therapeutic transducer, is positioned against the patient’s chest (panel (**a**)). The aortic valve is targeted and imaged using 2-D echo live monitoring (panel (**b**)). The image was reproduced with the permission of Cardiawave, Levallois-Perret, France.

The Valvosoft device is based on a new and unique ultrasound technology known as NIUT. This patented and distinctive ultrasound technology emits non-invasive high-intensity ultrasound focused on a target inside the body with high precision and accuracy. The ultrasound applicator, applied to the patient’s chest, delivers precisely focused and controlled short ultrasound pulses (<20 μs) on the calcified valve to produce non-thermal mechanical tissue softening of the stiffened calcified aortic valve. Echocardiographic live imaging, possible with a 2D echocardiographic imaging probe inserted in the center of the therapy transducer, allows valve movements to be followed in real time. The therapy is delivered by a multi-element transducer (bandwidth 700 kHz to 1.25 MHz), enabling electronic steering to position the focal point at different depths. Medio-lateral positioning of the focal point is performed by a custom-designed mechanical system. The combined echocardiographic live imaging, electronic steering, and lateral mechanical displacement of the focal point enable precise treatment of the aortic valve by the operator. Thereby, the operator can set the power of the delivered therapeutical ultrasound energy, which can be individually adapted and increased until the cavitation bubble cloud appears at the focal point. Further information is provided in Supplemental Videos S1 and S2 and Figure 1.

The treatment consisted of a maximum of 60 min of energy that was applied, divided into sessions of a maximum of 10 min, with short pauses of 1 to 5 min between the sessions. For each treatment session, the Valvosoft applicator was placed on the subject’s thorax and acoustically coupled with ultrasound coupling gel indicated for ultrasound therapy. Guided by the ultrasound images, the operator manually adjusted the applicator’s position to obtain an optimal aortic valve view. The therapy session was then started on the defined target zone after confirmation by the operator. Patients could be placed in a dorsal or left lateral decubitus position to optimize aortic valve view and applicator coupling with their thorax. Patients were treated under conscious sedation and with appropriate pain management, if necessary. An anesthesiologist was present in case general anesthesia was requested.

During the procedure, the following parameters were recorded and further analyzed for each patient: cumulative focal energy (J/mm^2^), mean of interface human machine/therapy gain (%), steering mean (mm), maximum and mean of peak acoustic power (W), maximum and mean of the intensity spatial-peak pulse-average (ISPPA; W/mm^2^), mean of the focal acoustic energy per mm^2^ (J/mm^2^), and target zone surface from computation (mm^2^). No specific medication regime was required.

The patients were hospitalized for one night to rule out serious adverse events and to obtain post-procedural cardiac enzymes.

Echocardiographic assessments are site-reported and were performed according to the guidelines of the American Society of Echocardiography [14]. The magnetic resonance imaging protocol has been described previously [11].

CRP was determined by immunoturbidimetry using the Architekt c8000 device (Abbott, Irving, TX, USA). Agglutination is detected as a change in absorbance (572 nm) proportional to the amount of CRP in the sample. The analytical sensitivity of the test is 0.5 mg/L. The reference value is <8 mg/L. High-sensitivity Troponin T was determined by electro-chemiluminescent immunoassay, using “ECL” technology with the cobas e 601 module (Roche Diagnostics, Mannheim Germany, manufacturer Hitachi High-Technologies Corporation, Tokyo, Japan), using the sandwich principle. The analytical sensitivity of the test is 3 ng/L, and the reference upper limit (99th percentile) is <14 ng/L. 

As a possible marker of hemodynamic alterations in the blood flow in valvular heart disease, including aortic valve shear stress [15], we assessed the von Willebrand factor (vWF). The hemostatic (adhesive) shear flow is able to stretch high-molecular-weight multimers (HMWs) and alter their conformation, unfolding it for better platelet, collagen, and factor VIII adherence but, at the same time, exposing it to proteolytic cleavage, resulting in a reduction in HMW multimers. These conformational changes in HMW multimers induced by shear flow are fast, and vWF responds within minutes to any significant change in hemodynamic valve status [16,17,18].

At the time of the study, we tested von Willebrand factor activity (VWF Ac) [19]. The activity was determined by immunoturbidimetry using the BCS XP apparatus (Siemens AG, Marburg, Germany). The INNOVANNCE^®^ VWF AC assay is a sensitive test for direct, WHO-standardized determination of vWF Ac. It allows one to mimic the way in which vWF binds to glyocoprotein Ib, the major vWF receptor protein on platelets. The analytical sensitivity is 2.2%, and the reference value is (% of normal) 48–173%. 

BNP was determined by chemiluminescent immunoassay using the Atellica IM analyzer (Siemens Healthcare Diagnostics Inc., Tarrytown, NY, USA). The test is a double-sandwich immunoassay, using direct chemiluminescent technology, which uses constant amounts of two monoclonal antibodies. The analytical sensitivity is 2 pg/mL, and the reference value (95th percentile) is <100 pg/mL.

### 2.3. Endpoints and Definitions

The endpoints are described in detail on ClinicalTrials.gov NCT04665596. The primary safety endpoint, procedure-related mortality up to 30 days post-procedure, other safety endpoints, and neurological outcomes have been reported previously [11]. In this report, we focus on the primary outcome of device performance to modify valve hemodynamic properties and left ventricular function, e.g., measured effects on the aortic valve mean pressure gradient and area and left ventricular ejection fraction. 

The longitudinal left ventricular function was assessed using left ventricular peak global longitudinal strain (GLS) measured with two-dimensional speckle-tracking echocardiography. Myocardial work parameters were determined to provide a more load-independent measure compared with left ventricular ejection fraction and GLS. The following parameters were assessed: the global work index (GWI) as measure for the myocardial work performed by the left ventricle during systole; the global constructive work (GCW) that assesses myocyte shortening and lengthening during systole and isovolumic relaxation, respectively; global wasted work (GWW) that assesses myocyte lengthening during systole and shortening during isovolumic relaxation, respectively; and global work efficiency (GWE), calculated as GCW/(GCW + GWW) [20]. GLS and left ventricular myocardial work parameters were measured using ECHOPAC version 204 (GE Healthcare, Chino, CA, USA) according to the methodology described by Jain et al. [20].

We also evaluated the effects of the therapy on stroke work loss (SWL) and valvuloarterial impedance (Zva), as well as the acoustic characteristics of focused ultrasound applied during the treatment, including peak acoustic power, mean acoustic energy, and ISPPA. SWL is the amount of left ventricular mechanical energy dissipated because of left ventricular obstruction: SWL = [mean pressure gradient/(mean pressure gradient + systolic arterial pressure)] × 100 [16]. Zva was calculated using the sum of systemic arterial pressure and mean pressure gradient divided by the stroke volume index: Zva = (systolic arterial pressure+ mean transvalvular pressure gradient)/stroke volume index [16]. Peak acoustic power is the peak acoustic power emitted by the therapy transducer, and the mean acoustic energy is the average acoustic power emitted by the therapy transducer per mm^2^ (of target zone) cumulated over the whole therapy time. ISPPA is defined as acoustic intensity at the focal spot averaged over a therapeutic pulse.

Acoustic characteristics of the focused ultrasound applied during the treatment, including peak acoustic power, mean acoustic energy, ISPPA, and target zone surface, were analyzed in terms of correlation with the changes in aortic valve and left ventricular properties.

### 2.4. Statistical Analysis

This is an exploratory pilot study; thus, no formal justification for the sample size was made. Enrollment of patients willing to undergo two brain magnetic resonance imaging scans (which was required per protocol) is very difficult in this multimorbid population. Still, the number of 10 patients was expected to provide robust evidence of the cerebral effect of the treatment and to provide hypothesis-generating first insights into the treatment effects. 

Categorical data are presented as numbers and percentages and continuous variables by means and standard deviations, medians, and interquartile ranges. Comparisons at different time points were tested with the related-samples Friedman’s two-way analysis of variance by ranks. The correlation analysis was performed using Spearman’s rho and using regression curve estimation models. No imputation on missing data was performed. Data were analyzed using IBM SPSS version 20.

## 3. Results

The baseline and procedural parameters are provided in Table 1 and Table 2, and the individual procedural data are provided in Supplemental Table S2. The median treatment time was 57 min (interquartile range: 51–60); eight patients reported pain that was resolved with pain medication.

**Figure 2:**
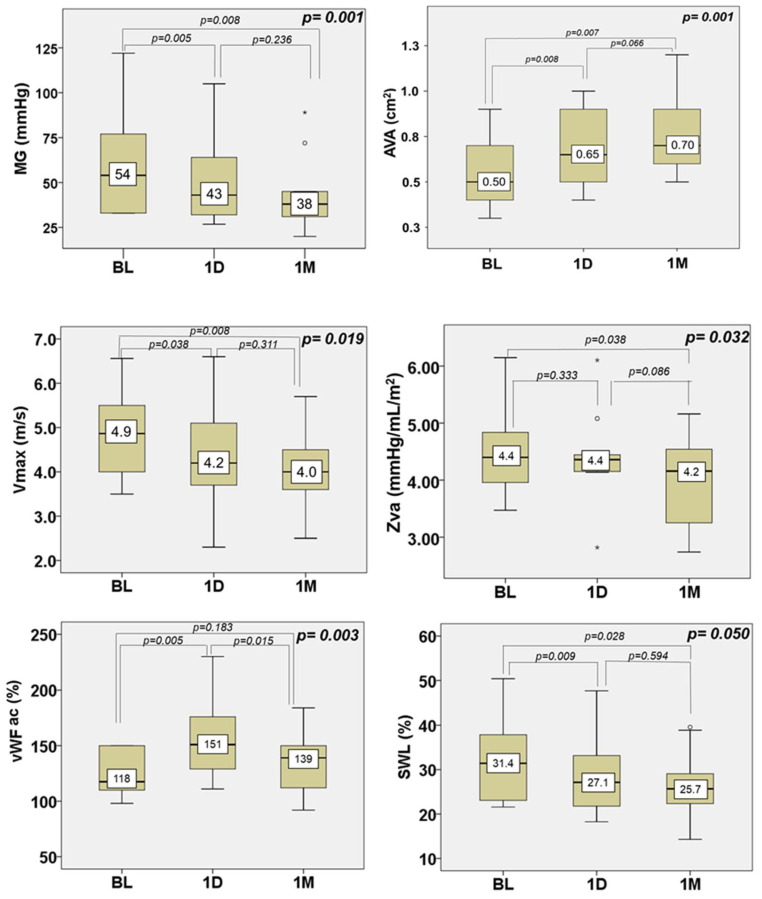
Changes in aortic valve hemodynamics, valvular shear stress, and stroke work loss between baseline, one day, and one month. The box represents the interquartile range (IQR), and the number represents the median. The whisker represents the maximum and minimum values, excluding extreme results that are marked as white circles (>1.5 times IQR) or asterisks (>3 times IQR). Comparisons between different time points were tested with related-samples Friedman’s two-way analysis of variance by ranks and *p* (in the upper right corners of the graphs) representing the comparison of baseline, BL, versus one day, 1D, versus one month, 1M. Comparisons between two time points were tested with related-samples Wilcoxon signed-rank test. *p*-values for each comparison are indicated. Aortic valve hemodynamics are presented by aortic valve area (AVA), mean pressure gradient (MG), peak velocity (Vmax), and valvular–arterial impedance (Zva), and the valve shear stress is presented by the von Willebrand factor (vWF ac). The energy the left ventricle dissipates due to aortic stenosis was estimated by stroke work loss (SWL).

Evaluating aortic valve hemodynamics and shear stress, we observed a progressive increase in aortic valve area (AVA) following NIUT treatment. AVA rose by 39%, from a median of 0.5 cm^2^ at baseline to 0.65 cm^2^ at one day and 0.7 cm^2^ at one month, *p* = 0.001. Similarly, there was a significant decrement in mean gradient by 23% (from 54 mmHg to 43 mmHg to 38 mmHg, *p* = 0.001), in peak velocity (Vmax 4.9 m/s vs. 4.2 m/s vs. 4.0 m/s, *p* = 0.019), and in valvuloarterial impedance (*p* = 0.032); the SWL decreased by an amount that was nearly significant (*p* = 0.050), whereas there was no effect on aortic regurgitation (Figure 1, individual data in Supplemental Tables S3 and S4). The vWF Ac increased significantly on day 1 but returned to similar values to baseline at one month (Figure 2, individual data in Supplemental Table S5).

**Figure 3:**
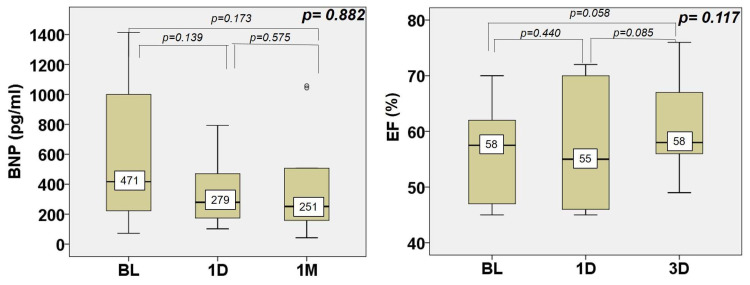
Changes in left ventricular function and left ventricular wall shear stress between baseline, one day, and one month. See legend in Figure 2. Left wall shear stress is represented by brain natriuretic peptide (BNP) assessment. EF = ejection fraction.

**Figure 4:**
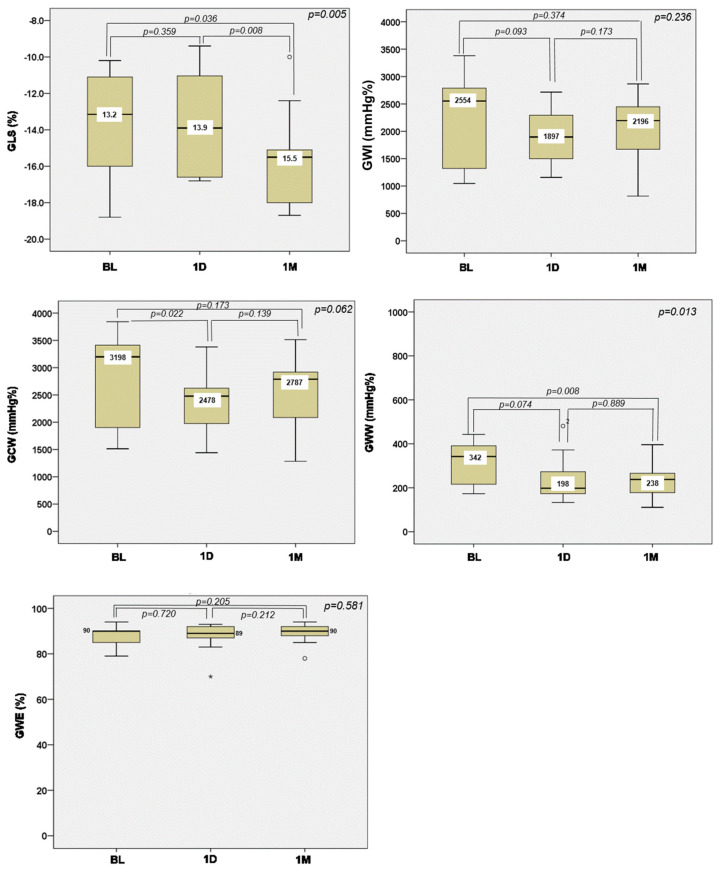
Changes in left ventricular myocardial mechanics between baseline, one day, and one month after procedure. See legend in Figure 2. GCW = global constructive work, GLS = global longitudinal strain, GWE = global work efficiency, GWI = global work index, GWW = global wasted work.

In terms of left ventricular function, the left ventricular ejection fraction did not change, but the BNP as a marker of left ventricular wall stress dropped from a median of 417 pg/mL at baseline to 251 pg/mL at one month (*p* = 0.882) (Figure 3). Assessing the left ventricular myocardial mechanics, GLS significantly increased from a median of |−13.2|% at baseline to |−15.5|% at one month (*p* = 0.036), and GWI, GCW, and GWW decreased with the GWW change, reaching statistical significance (from a median of 342 mmHg% at baseline to a median of 238 mmHg% at one month, *p* = 0.008) (Figure 4, individual data in Supplemental Table S6).

**Figure 5:**
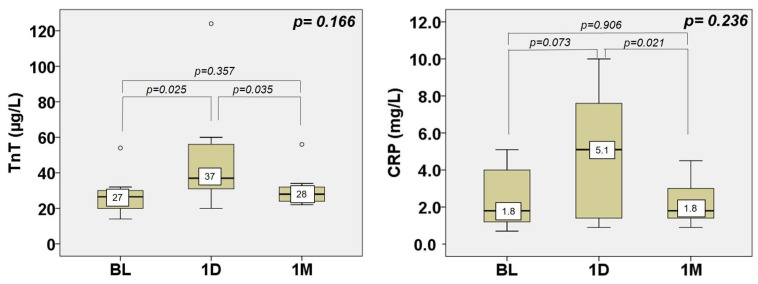
Changes in biomarkers of myocardial injury (high-sensitivity Troponin T) and systematic inflammatory response between baseline, one day, and one month. See legend in Figure 2. TnT = high-sensitivity Troponin T, CRP = C-reactive protein.

Hs-TnT, a biomarker for myocardial injury, was increased at baseline and additionally rose on the first day, returning to the baseline values after the first month. Specifically, it raised from a median of 27 µg/L at baseline to 37 µg/L on day one and dropped to 28 µg/L at one month again. A parallel rise and drop of CRP accompanied the hs-TnT rise and fall. Neither were accompanied by ECG changes or subjective complaints suggestive of myocardial injury (Figure 5, individual data in Supplemental Table S5).

**Figure 6:**
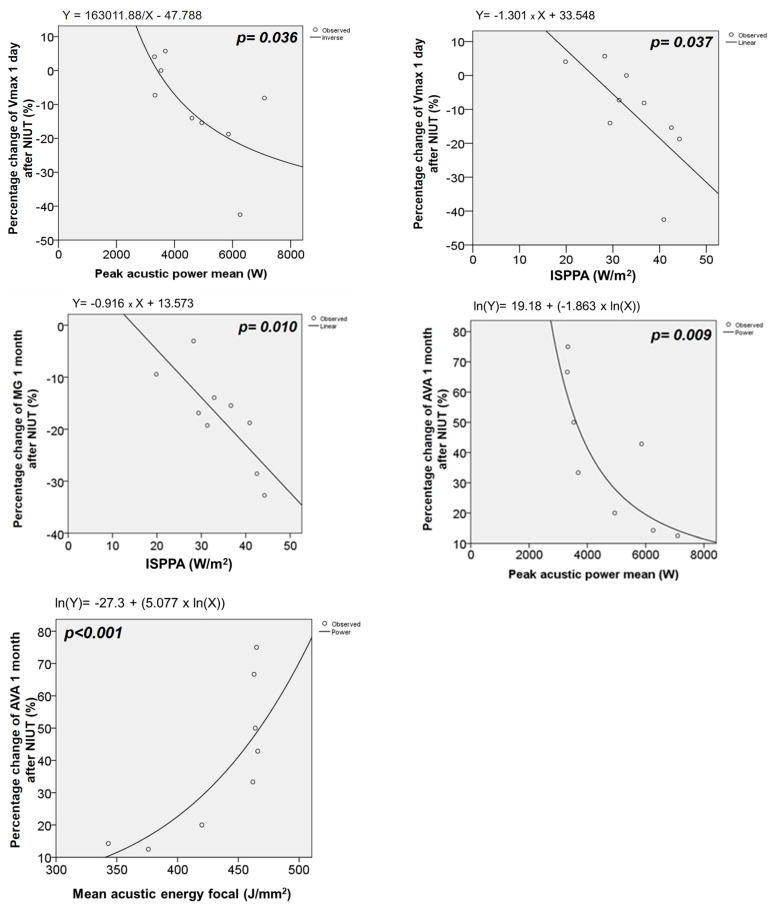
Relationship between procedural parameters and aortic valve hemodynamics. The curves represent further details of the significant relationship between procedural and aortic valve hemodynamics. The calculations were performed using regression curve estimation models. The circles represent observations; the curve is the calculated curve that best fits. AVA = aortic valve area, ISPPA = intensity spatial-peak pulse-average. MG = mean pressure gradient, NIUT = non-invasive ultrasound therapy.

To better understand the relationship between procedural parameters and percent changes (Δ) in aortic valve hemodynamics, regression models with curve estimations were performed to determine the best fit model. Inverse correlations were observed between the change in Vmax at one day and procedural peak acoustic power and ISPPA, as well as between the changes in mean gradient and SWL at one day with the ISPPA. The AVA change at one month had a negative correlation with the peak acoustic power and a positive correlation with the acoustic energy focal, whereas one-month-change in the valvuloarterial impedance had a negative correlation with ISPPA (Table 3, Figure 6). The remaining procedural parameters did not correlate with outcomes, although this might be attributed to the small number of patients.

The New York Heart Association (NYHA) class and quality of life improved at follow-up, as depicted in Supplemental Table S7.

## 4. Discussion

The main findings of this analysis are that in patients with severe aortic stenosis, there is a positive effect of NIUT on (a) aortic valve hemodynamics and valve shear stress and on (b) the left ventricle, with improved GLS and myocardial work parameters; in addition, (c) the procedure is safe, with only a transient and clinically non-significant response of laboratory parameters indicative of myocardial injury that returned to baseline values at one month.

### 4.1. Effect of NIUT on Aortic Valve Hemodynamics and Valve Shear Stress

We observed a significant increase in AVA (by 39% at one month) and a significant decrease in mean gradient, peak velocity (by 23% and 14%, respectively), valvuloarterial impedance, and stroke work loss, confirming the efficacy of NIUT applied with the Valvosoft device. Likewise, the multi-center first-in-human series reported significant improvement in AVA, mean gradient, and valvular impedance through six months [12]. While this improvement in hemodynamic parameters did not resolve aortic stenosis, it reduced it, resulting in a decrease in New York Heart Association class by 1.2 classes and an increase in quality of life by 22%, as reported previously [11]. Moreover, there was a near significant reduction in valvuloarterial impedance and SWL post-procedure; both parameters are known to be predictors of left ventricular dysfunction and adverse outcomes after aortic valve replacement [21]. Likewise, the peak velocity is a prognostic factor. It was reduced from a baseline value of 4.9 m/s, that nearly indicates *very* severe aortic stenosis (threshold of ≥5.0 m/s for very severe aortic stenosis), to 4.0 m/s, a reduction which is known to impact event-free survival [22].

It is relevant that there was no increase in aortic regurgitation, whereas in initial surgical studies with ultrasound therapy, the therapy led to an unacceptable increase in aortic regurgitation at follow-up (severe aortic regurgitation in 26% of cases, and moderate aortic regurgitation in 37% of cases) [23].

Procedural parameters impacted the hemodynamic outcomes. Peak acoustic power and ISPPA already had a significant inverse correlation with the peak velocity at one day, which disappeared at one month. The same pattern was observed for the correlation of ISPPA and the reduction in mean pressure gradient and SWL. On the contrary, AVA and valvuloarterial impedance significantly correlated with procedural parameters but only after one month, which could be a sign that perhaps more time is needed for structural changes to occur in proportion to the energy applied during the treatment. However, this assumption is speculative, and outcomes could be merely related to the small sample size.

Especially interesting is that one-month AVA increase had an *inverse* relation with peak acoustic power but a *positive* relation with the mean acoustic energy focal. It seems that the softening of the aortic valve with an AVA increase is proportional to the cumulative acoustic energy delivered at the focal level (energy per mm^2^) but that there is a paradoxical reaction if the peak acoustic power (energy per time) is above a certain value; this will not result in a larger AVA increase but a rather smaller AVA increase. With the caveats that this observation is only based on 10 patients and the fact that effects at the focal point were not measured, it might be a sign that the cumulative acoustic energy is important, but the delivery *rate* (not too fast) is also important, as the cavitation process within the calcified aortic valve needs time to develop properly during the treatment.

In terms of vWF, there are several ways to measure this parameter [16]. We used a recently introduced new vWF test method and, hence, did not use the same methodology described as correlated with valve shear stress and with aortic valve stenosis [16,18,24]. However, there are some assumptions that might provide a hint that vWF Ac is also correlated with valve shear stress. First, there are reports that vWF Ac is negatively correlated with mean pressure gradient and peak velocity [25]. Second, similar to Smadja et al., who observed an increase in HMW multimers at day one after NIUT [18], we observed a significant increase in vWF Ac activities at day one after NIUT. Third, there was a similar, significant raise of HMW vWF multimers and vWF Ac reported three to five days after surgical aortic valve replacement [26]. It could thus be assumed that HMW vWF multimers and vWF Ac both react on alterations in valve shear stress. However, due to the low number of patients, and given the fact that vWF Ac values were within the normal ranges at all time points, our results should not be over-interpreted.

### 4.2. Impact of NIUT on the Left Ventricle

There was no significant increase in left ventricular ejection fraction between baseline and one month, which is not surprising considering that the baseline values were within the normal ranges. The drop in the BNP and improvement in GLS and myocardial work are of interest though. While it was not statistically significant, there was a numerical BNP decrease by nearly half and a decrease in scattering around the median. The main stimulus for the BNP synthesis is cardiac wall stress [27], and BNP is a key parameter that is correlated with left end-diastolic wall stress [28]. Thus, it seems that the stress might slightly decrease after NIUT due to partial deliberation of the aortic valve and unloading of the left ventricle. 

Left ventricular GLS is a more sensitive marker than the left ventricular ejection fraction to detect early improvement in left ventricular systolic function after TAVR in patients with severe aortic stenosis [29]. In our series, we observed a significant GLS recovery one month after NIUT, which further supports the left ventricular response, even to incomplete unloading achieved by NIUT. Importantly, the median value of |−15.5|% for GLS one month after NIUT is above the threshold of |−15|% that has been associated with poor outcomes in patients with severe aortic stenosis and preserved left ventricular ejection fraction [30].

In addition, we observed an improvement in myocardial work parameters. Studies showed a significant reduction in GWI and GCW after TAVR in patients with severe aortic stenosis [20,31]. In our study, GWI, GCW, and GWW reduced after NIUT (statistical significance was only reached for GWW). This might suggest that with the partial left ventricular unloading as a result of the NIUT, the left ventricle manages to work more efficiently, which would be in concordance with the near-significantly decreased SWL.

### 4.3. Safety

As reported previously, the systematic pre- and post-procedural magnetic resonance imaging did not reveal any ischemic changes, and the Mini Mental State Examination results remained stable. Furthermore, no death, major adverse event, or cerebrovascular event occurred within one month [11].

Laboratory parameters indicative for myocardial injury (hs-TnT) and systematic inflammatory response (CRP) increased on day one compared to baseline but decreased again at one month. The change between baseline and one month was not statistically significant, but there was a statistically significant, but clinically not relevant, increase in hs-TnT at one day. In terms of CRP, the peak CRP value was 5.1 mg/L, thus still within the normal range, confirming the safety of the procedure. In terms of hs-TnT, the threshold for myocardial injury in TAVR is an 18.3-fold increase above the upper limit of normal [32], which would be 256 µg/L, a value that was not reached by any of the patients. The slight hs-TnT rise did not even meet any of the criteria of peri-procedural myocardial injury for percutaneous coronary interventions, which is a less-invasive procedure than TAVR [33]. 

These safety parameters, along with the histopathological outcomes from animal studies [5,10], and the reported absence of any adverse event related to the procedure in the multi-center first-in-human series [12], reflect that NIUT is non-invasive and can be safely applied even in old and multimorbid patients. Moreover, these data are relevant when considering the use of ultrasound to crack calcium as proposed in younger patients to retard TAVR or surgical valve replacement or to facilitate TAVR [6,7,8].

This study has the inherent limitations of a small case series of 10 patients. Hence, outcomes can only be interpreted as hypothesis-generating and require confirmation in larger trials. The assessment of further vWF markers, such as vWF HMW multimers, would have been useful to compare our results to other treatment modalities. 

While one-month data are important, e.g., to stabilize patients prior to a more aggressive and more effective procedure like TAVR, the short interval makes it difficult to determine whether improvements are long-term or reversible; one-year data are awaited. Furthermore, a pivotal study is ongoing to assess patients that might potentially benefit best from the treatment (e.g., patients with moderate aortic stenosis) and to further investigate best treatment practices.

## 5. Conclusions

This first-in-human cohort thoroughly investigated the biological effects of NIUT. It confirmed that NIUT is a safe and effective therapy with significant effects on hemodynamic parameters that are proportional to treatment parameters with positive outcomes in terms of valvular shear stress, left ventricular wall stress and myocardial mechanics, without significant negative effects on biological markers of myocardial injury and inflammation. These results are hypothesis-generating and need to be confirmed in larger trials. Valvosoft NIUT may serve as a bridge to other severe aortic stenosis treatments or as a therapy in patients that cannot even undergo TAVR treatment. It is hence seen as complementary to existing treatment modalities. 

## Figures and Tables

**Table 1:** Baseline characteristics.

	N = 10
Age (years)	80.0 (74.5–83.0)
Female	6 (60%)
Male	4 (40%)
Systemic hypertension	10 (100%)
Coronary artery disease	7 (70%)
Angina	4 (40%)
Diabetes mellitus	4 (40%)
Renal insufficiency	4 (40%)
Peripheral artery disease	4 (40%)
Prior cerebrovascular event	1 (10%)
New York Heart Association	
2	4 (40.0%)
3	6 (60.0%)
4	0 (0.0%)
STS score (%)	6.7 (4.6–8.2)

Data are displayed as median (Interquartile range) and n (%).

**Table 2:** Procedural characteristics.

	N = 10
Cumulative focal energy (J/mm^2^)	463 (387–465)
Gain IHM mean (%)	52 (48–58)
Steering mean (mm)	122 (113–130)
Peak acoustic power max (W)	8506 (7317–8922)
Peak acoustic power mean (W)	4143 (3379–5628)
ISPPA max (W/mm^2^)	58 (58–58)
ISPPA mean (W/mm^2^)	33 (30–40)
Mean acoustic energy focal (J/mm^2^)	463 (387–465)
TZ surface (from computation, mm^2^)	125 (110–166)

Data are displayed as median (interquartile range) and n (%). IHM = interface human machine/therapy gain (one of the parameters that defines the level of energy of the delivered therapeutic ultrasound on the focal point), ISPPA = intensity spatial-peak pulse-average, TZ = target zone.

**Table 3:** Relationship between procedural parameters and percent change in aortic valve hemodynamics at one day and one month.

	Peak Acoustic Power Mean (W)		I_SPPA_ Mean (W/mm^2^)		Mean Acoustic Energy Focal (J/mm^2^)	
	Correlation Coefficient	*p*	Correlation Coefficient	*p*	Correlation Coefficient	*p*
**∆ 1D**						
Vmax (m/s)	**−0.697**	**0.025**	**−0.794**	**0.006**	0.321	0.365
MG (mmHg)	−0.504	0.138	**−0.745**	**0.013**	−0.030	0.934
AVA (cm^2^)	−0.232	0.519	0.030	0.933	0.311	0.382
SWL (%)	−0.309	0.385	**−0.673**	**0.033**	−0.188	0.603
Zva (mmHg/mL/m^2^)	0.030	0.934	−0.188	0.603	0.176	0.627
**∆ 1M**						
Vmax (m/s)	−0.367	0.332	−0.433	0.244	0.350	0.356
MG (mmHg)	−0.017	0.966	−0.067	0.865	−0.017	0.066
AVA (cm^2^)	**−0.733**	**0.025**	−0.517	0.154	**0.700**	**0.0036**
SWL (%)	0.100	0.798	0.200	0.606	0.283	0.460
Zva (mmHg/mL/m^2^)	−0.5678	0.112	**−0.700**	**0.036**	−0.100	0.798

The correlation analysis was performed using Spearman’s rho. ∆ = delta, AVA = aortic valve area, MG = mean pressure gradient, SWL = stroke work loss, Zva = valvuloarterial impedance.

## Data Availability

The original data presented in the study are included in the article, and further inquiries can be directed to the corresponding author.

## Supplementary Materials

The following supporting information can be downloaded at: https://www.mdpi.com/article/10.3390/jcm13164607/s1, Table S1: Inclusion and Exclusion criteria, Table S2: Procedural characteristics, Table S3: Echocardiographic assessments at baseline, one day and one month, Table S4: Change in aortic valve parameters at one day and one month post-procedure, Table S5: Laboratory assessments at baseline, one day, and one month, Table S6. Left ventricular myocardial mechanic assessed by two-dimensional speckle-tracking at baseline, one day and one month, Table S7. Patient assessments at baseline and one month, Video S1: Mechanism of Non-Invasive Ultrasound Therapy (courtesy of Cardiawave), Video S2: Cavitation, Video S3: Aortic valve opening in patient with severe aortic stenosis before the treatment (echocardiography, parasternal long axis), Video S4: Aortic valve opening in patient with severe aortic stenosis after the treatment (echocardiography, parasternal long axis).
