# Supplementary material for: Non-Invasive Ultrasound Therapy for Severe Aortic Stenosis: Early Effects on the Valve, Ventricle, and Cardiac Biomarkers (A Case Series)
Source: J Clin Med. 2024 Aug 7;13(16):4607. doi: 10.3390/jcm13164607 (PMC11354631; doi:10.3390/jcm13164607)
Supplement: Supplementary file 1 [file jcm-13-04607-s001.zip › 240707 Valvosoft Supplement-clean.docx]

Non-invasive ultrasound therapy for severe aortic stenosis: early effects on the valve, ventricle, and cardiac biomarkers (a case series)

**Supplemental Table S1: Inclusion and Exclusion Criteria**

| Inclusion Criteria | 1. Subjects suffering from severe symptomatic aortic valve stenosis according to ESC 2017 definition, including subjects with a bicuspid valve. 2. Patient is not eligible for TAVR/SAVR according to local Heart Team. 3. Age ≥18 years. 4. Subjects who are willing to provide a written informed consent prior to participating in the study. 5. Subjects who can comply with the study follow up or other study requirements. |
| --- | --- |
| Exclusion Criteria | 1. Subjects with any electrical device implanted. 2. Subjects with unstable arrhythmia not controlled by medical treatment. 3. Subjects with implanted mechanical valve in any position or bio prosthetic valve in aortic position. 4. Subjects with complex congenital heart disease. 5. Chest deformity. 6. Cardiogenic shock. 7. History of heart transplant. 8. Subjects requiring other cardiac surgery procedures (bypass graft surgery, mitral valve procedure, tricuspid valve procedure) within one month after treatment. 9. Thrombus in the heart. 10. Acute myocardial infarction (MI), stroke or transient ischemic attack (TIA) within one month prior to enrolment. 11. Subjects who are pregnant or nursing. 12. Subjects who are participating in another research study for which the primary endpoint has not been reached. |

ESC-European Society of Cardiology, SAVR-surgical aortic valve replacement, TAVR-transcatheter aortic valve replacement

**Supplemental Table S2: Procedural characteristics**

| **Patient** | **Cumulative focal energy (J/mm²)** | **Gain IHM mean (%)** | **Steering mean (mm)** | **Peak Acoustic Power max (W)** | **Peak Acoustic Power mean (W)** | **I_SPPA_ max (W/mm²)** | **I_SPPA_ mean (W/mm²)** | **Mean Acoustic Energy Focal (J/mm²)** | **TZ Surface (from computation, mm²)** |
| --- | --- | --- | --- | --- | --- | --- | --- | --- | --- |
| 1 | 343 | 57 | 129 | 9120 | 6258 | 58 | 41 | 343 | 139 |
| 2 | 376 | 58 | 141 | 9048 | 7097 | 58 | 37 | 376 | 96 |
| 3 | 306 | 48 | 130 | 8543 | 4600 | 58 | 29 | 306 | 138 |
| 4 | 420 | 58 | 116 | 8543 | 4939 | 58 | 43 | 420 | 149 |
| 5 | 462 | 47 | 121 | 8469 | 3686 | 58 | 28 | 462 | 142 |
| 6 | 463 | 40 | 133 | 6986 | 3313 | 53 | 20 | 463 | 114 |
| 7 | 464 | 51 | 112 | 7552 | 3533 | 58 | 33 | 464 | 108 |
| 8 | 465 | 50 | 111 | 7239 | 3328 | 58 | 31 | 465 | 60 |
| 9 | 466 | 60 | 122 | 9120 | 5858 | 58 | 44 | 466 | 193 |
| 10 | 467 | 53 | 94 | 3733 | 2731 | 41 | 33 | 467 | 207 |
| Mean | 423 | 52 | 121 | 7835 | 4534 | 56 | 34 | 423 | 136 |
| SD | 60 | 6 | 13 | 1638 | 1467 | 5 | 7 | 60 | 44 |
| Median | 463 | 52 | 122 | 8506 | 4143 | 58 | 33 | 463 | 125 |
| IQR | 387–465 | 48–58 | 113–130 | 7317–8922 | 3379–5628 | 58–58 | 30–40 | 387–465 | 110–166 |

IHM= = interface human machine / therapy gain (one of the parameters that defines the level of energy of the delivered therapeutic ultrasound on the focal point), I_SPPA_=Intensity Spatial Peak Pulse Average, TZ=target zone

**Supplemental Table S3: Echocardiographic assessments at baseline, one day and one month**

| Patient | AVA (cm2) | | | MG (mmHg) | | | Vmax (m/sec) | | | AR (+) | | | LVEF (%) | | | SVi (ml/m²) | | | SWL (%) | | | Zva (mmHg/mL/m²). | | |
| --- | --- | --- | --- | --- | --- | --- | --- | --- | --- | --- | --- | --- | --- | --- | --- | --- | --- | --- | --- | --- | --- | --- | --- | --- |
|  | BL | 1D | 1M | BL | 1D | 1M | BL | 1D | 1M | BL | 1D | 1M | BL | 1D | 1M | BL | 1D | 1M | BL | 1D | 1M | BL | 1D | 1M |
| 1 | 0.7 | 0.8 | 0.8 | 33 | 27 | 28 | 4.0 | 2.3 | 2.5 | 1 | 1 | 1 | 45 | 45 | 49 | 33.5 | 27.2 | 32.3 | 23.1 | 18.3 | 20.3 | 3.5 | 4.4 | 3.3 |
| 2 | 0.8 | 0.9 | 0.9 | 33 | 28 | 31 | 3.7 | 3.4 | 3.6 | 2 | 1.5 | 1.5 | 45 | 46 | 57 | 29.9 | 35.9 | 37.9 | 22.3 | 21.8 | 23.7 | 3.95 | 2.8 | 2.7 |
| 3^(1)^ | 0.4 | 0.5 | - | 77 | 64 | - | 5.7 | 4.9 | - | 1 | 1.5 | - | 63 | 72 | - | 34.8 | 33.2 | - | 39.1 | 39.0 | - | 4.51 | 4.1 | - |
| 4 | 0.5 | 0.6 | 0.6 | 70 | 50 | 45 | 5.2 | 4.4 | 4.2 | 1.5 | 1.5 | 1.5 | 62 | 70 | 76 | 26.3 | 22.4 | 27.8 | 37.8 | 29.4 | 29.0 | 6.2 | 6.1 | 4.6 |
| 5 | 0.9 | 0.9 | 1.2 | 33 | 32 | 20 | 3.5 | 3.7 | 3.0 | 1.5 | 1.5 | 1.5 | 60 | 70 | 69 | 31.9 | 28.2 | 26.2 | 21.6 | 21.8 | 14.3 | 4.0 | 4.4 | 4.3 |
| 6 | 0.3 | 0.4 | 0.5 | 53 | 48 | 41 | 4.9 | 5.1 | 4.5 | 1 | 1 | 1 | 58 | 55 | 56 | 27.5 | 28.0 | 25.1 | 34.6 | 32.4 | 29.1 | 4.8 | 4.3 | 4.5 |
| 7 | 0.4 | 0.4 | 0.6 | 122 | 105 | 89 | 6.6 | 6.6 | 5.7 | 2 | 2 | 2 | 47 | 46 | 49 | 40.9 | 35.1 | 37.0 | 50.4 | 47.7 | 38.9 | 5.3 | 5.1 | 5.2 |
| 8 | 0.4 | 0.7 | 0.7 | 83 | 67 | 72 | 5.5 | 5.1 | 5.2 | 2 | 2 | 2 | 54 | 49 | 58 | 40.5 | 38.7 | 43.9 | 36.9 | 33.2 | 39.56 | 4.6 | 4.4 | 3.5 |
| 9 | 0.7 | 1.0 | 1.0 | 55 | 37 | 38 | 4.8 | 3.9 | 4.0 | 0 | 0 | 0 | 70 | 72 | 63 | 38.7 | 31.0 | 42.0 | 28.2 | 21.5 | 25.68 | 4.2 | 4.2 | 2.7 |
| 10 | 0.5 | 0.6 | 0.7 | 42 | 38 | 36 | 4.1 | 4.0 | 3.9 | 1.5 | 1.5 | 2 | 57 | 55 | 67 | 31.2 | 29.2 | 31.5 | 25.2 | 24.8 | 22.36 | 4.3 | 4.3 | 4.2 |
| Mean | 0.56 | 0.65 | 0.78 | 60 | 50 | 44 | 4.8 | 4.3 | 4.1 | 1.4 | 1.4 | 1.4 | 56 | 58 | 60 | 33.5 | 30.9 | 33.7 | 31.9 | 29.0 | 27.0 | 4.5 | 4.4 | 3.9 |
| SD | 0.20 | 0.21 | 0.22 | 29 | 24 | 22 | 1.0 | 1.2 | 1.0 | 0.59 | 0.55 | 0.61 | 8 | 12 | 9 | 5.2 | 4.9 | 6.8 | 9.4 | 9.3 | 8.3 | 0.8 | 0.8 | 0.9 |
| Median | 0.5 | 0.65 | 0.7 | 54 | 43 | 38 | 4.9 | 4.2 | 4.0 | 1.5 | 1.5 | 1.5 | 58 | 55 | 58 | 32.7 | 30.1 | 32.3 | 31.4 | 27.1 | 25.7 | 4.4 | 4.4 | 4.2 |
| IQR | 0.4–0.7 | 0.5-0.9 | 0.6–0.9 | 33–79 | 31-65 | 30–59 | 3.9–5.5 | 3.6-5.1 | 3.3–4.9 | 1–2 | 1–2 | 1–2 | 49–62 | 46-71 | 53–68 | 29.3-39.2 | 27.8-35.3 | 27.0-40.0 | 22.9-38.2 | 21.7-34.6 | 21.3-34.0 | 4.0-4.9 | 4.1-4.6 | 3.0-4.6 |

^1^ Visit cancelled due to COVID-19. AR=aortic regurgitation, AVA=aortic valve area, D=day, LVEF=left ventricular ejection fraction, M=month, MG=mean pressure gradient, SVi=stroke volume index, SWL=stroke work loss, Zva=valvuloarterial impedance

**Supplemental Table S4: Change in aortic valve parameters at one day and one month post-procedure**

|  | AVA  (cm2) | | MG  (mmHg) | | Vmax  (m/sec) | | vWF Ac  (%) | | SVi  (mL/m2) | | SWL  (%) | | Zva  (mmHg/mL/m^2^) | |
| --- | --- | --- | --- | --- | --- | --- | --- | --- | --- | --- | --- | --- | --- | --- |
| Patient | ∆ 1D (%) | ∆ 1M (%) | ∆ 1D (%) | ∆ 1M (%) | ∆ 1D (%) | ∆ 1M (%) | ∆ 1D (%) | ∆ 1M (%) | ∆ 1D (%) | ∆ 1M (%) | ∆ 1D (%) | ∆ 1M (%) | ∆ 1D (%) | ∆ 1M (%) |
| 1 | 14.3 | 14.3 | -18.8 | -15.2 | -42.5 | -37.5 | 11.8 | 8.2 | -18.8 | -3.6 | -20.9 | -12.1 | 27.2 | -6.4 |
| 2 | 12.5 | 12.5 | -15.5 | -6.1 | -8.1 | -2.7 | 26.7 | 15.8 | 20.1 | 26.8 | -2.2 | 6.1 | -28.6 | -30.5 |
| 3^(1)^ | 25.0 | - | -16.9 | - | -14.0 | - | 31.6 | - | -4.6 | - | -0.2 | - | -8.3 |  |
| 4 | 20.0 | 20.0 | -28.6 | -35.7 | -15.4 | -19.2 | 2.9 | 8.7 | -14.8 | 5.7 | -22.3 | -23.3 | -0.7 | -25.1 |
| 5 | 0.0 | 33.3 | -3.0 | -39.4 | 5.7 | -14.3 | 53.3 | 22.7 | -11.6 | -17.9 | 0.9 | -33.8 | 10.8 | 8.9 |
| 6 | 33.3 | 66.7 | -9.4 | -22.6 | 4.1 | -8.2 | 17.3 | 2.0 | 1.8 | -8.7 | -6.4 | -16.2 | -10.4 | -6.1 |
| 7 | 0.0 | 50.0 | -13.9 | -27.1 | .00 | -13.6 | 13.3 | -6.1 | -14.2 | -9.5 | -5.3 | -22.9 | -3.5 | -1.9 |
| 8 | 75.0 | 75.0 | -19.3 | -13.3 | -7.3 | -5.5 | 45.8 | -3.7 | -4.4 | 8.4 | -10.1 | 7.2 | -4.1 | -23.8 |
| 9 | 42.9 | 42.9 | -32.7 | -30.9 | -18.8 | -16.7 | 30.0 | .00 | -19.9 | 8.5 | -23.7 | -9.0 | -0.6 | -34.5 |
| 10 | 20.0 | 40.0 | -9.5 | -14.3 | -2.4 | -4.9 | 12.2 | -2.6 | -6.4 | 1.0 | -1.2 | -11.1 | 0.9 | -3.0 |
| Mean | 24.3 | 39.4 | -16.8 | -22.7 | -9.9 | -13.6 | 24.5 | 5.0 | -7.3 | 1.2 | -9.13 | -12.8 | -1.7 | -13.6 |
| SD | 22.3 | 22.1 | 8.9 | 11.3 | 14.1 | 10.6 | 16.1 | 9.7 | 11.8 | 13.1 | 9.65 | 13.4 | 14.3 | 15.1 |
| Median | 20.0 | 40.0 | -16.17 | -22.6 | -7.69 | -13.63 | 22.00 | 2.00 | -9.0 | 1.0 | -5.9 | -12.1 | -2.1 | -6.4 |
| IQR | 9.4–35.7 | 17.1–58.3 | -21.6– -9.50 | -33.3– -13.8 | -16.2–1.0 | -18.0–5.2 | 12.1–35.1 | -3.2 12.3 | -15.8– -2.9 | -9.1– 8.5 | -21.2– -1.0) | -23.1 – -1.4 | -8.9–3.4 | -27.8– -2.4 |

^1^ Visit cancelled due to COVID-19. Delta (∆) is the percent change of parameters at one day (1D) or at one month (1M) compared to baseline. Negative ∆ represent at decrease. AVA=aortic valve area, D=day, M=month, MG=mean pressure gradient, SVi=stroke volume index, SWL=stroke work loss, vWF Ac=von Willebrand factor activity, Zva=valvuloarterial impedance

**Supplemental Table S5. Laboratory assessments at baseline, one day, and one month**

| Patient | BNP (pg/ml) | | | Troponin-T (µg/L)) | | | vWF activity (%) | | | CRP (mg/L) | | |
| --- | --- | --- | --- | --- | --- | --- | --- | --- | --- | --- | --- | --- |
|  | BL | 1D | 1M | BL | 1D | 1M | BL | 1D | 1M | BL | 1D | 1M |
| **1** | 223 | 174 | 251 | 26 | 31 | 24 | 110 | 123 | 119 | 1.2 | 0.9 | 1.4 |
| **2** | 258 | 246 | 158 | 23 | 20 | 22 | 120 | 152 | 139 | 4 | 5.9 | 2.3 |
| **3**^(1)^ | 521 | 279 | - | 20 | 40 | - | 114 | 150 |  | 1.7 | 1.4 |  |
| **4** | 313 | 428 | 328 | 27 | 124 | 32 | 138 | 142 | 150 | 1.1 | 5 | 1.6 |
| **5** | 114 | 133 | 115 | 20 | 31 | 24 | 150 | 230 | 184 | 4.1 | 5.2 | 3.4 |
| **6** | 854 | 470 | 507 | 54 | 56 | 56 | 150 | 176 | 153 | 1.9 | 10 | 3 |
| **7** | 1414 | - | 1043 | 14 | 46 | 34 | 98 | 111 | 92 | 0.7 | 9.2 | 0.9 |
| **8** | 1000 | 793 | 1058 | 32 | 28 | 32 | 107 | 156 | 103 | 2.8 | 7.6 | 4.5 |
| **9** | 72 | 102 | 42 | 30 | 34 | 28 | 150 | 195 | 150 | 1.7 | 1.4 | 1.8 |
| **10** | 1032 | 491 | 239 | 29 | 60 | 27 | 115 | 129 | 112 | 5.1 | 4.8 | 1.2 |
| Mean | 580 | 346 | 416 | 28 | 47 | 31 | 125 | 156 | 134 | 2.4 | 5.1 | 2.2 |
| SD | 463 | 221 | 384 | 111 | 30 | 10 | 20 | 36 | 29 | 1.5 | 3.2 | 1.2 |
| Median | 417 | 279 | 251 | 27 | 37 | 28 | 118 | 151 | 139 | 1.8 | 5.1 | 1.8 |
| IQR | 196-10080 | 154-481 | 137-775 | 20-31 | 30-57 | 24-33 | 109-1500 | 128-181 | 108-152 | 1.2-4.0 | 1.4-8.0 | 1.3-3.2 |

^1^ Visit cancelled due to COVID-19. BNP=brain natriuretic peptide, CRP=C-reactive protein, D=day, M=month, vWF=von Willebrand factor

**Supplemental Table S6. Left ventricular myocardial mechanic assessed by two-dimensional speckle-tracking at baseline, one day and one month**

| Patient | GLS  (%) | | | GWI  (mmHg%) | | | GCW  (mmHg%) | | | GWW  (mmHg%) | | | GWE  (%) | | |
| --- | --- | --- | --- | --- | --- | --- | --- | --- | --- | --- | --- | --- | --- | --- | --- |
|  | BL | 1D | 1M | BL | 1D | 1M | BL | 1D | 1M | BL | 1D | 1M | BL | 1D | 1M |
| 1 | -10.2 | -9.4 | -10.0 | 1321 | 1196 | 816 | 1902 | 1526 | 1284 | 400 | 481 | 396 | 81 | 70 | 78 |
| 2 | -11.2 | -11.0 | -15.4 | 1139 | 1158 | 1485 | 1513 | 1441 | 1776 | 216 | 133 | 150 | 85 | 87 | 91 |
| 3^(1)^ | -15.7 | -16.6 | - | 2737 | 2652 | - | 3171 | 2618 | - | 175 | 188 | - | 94 | 92 | - |
| 4 | -18.0 | -16.0 | -18.0 | 3381 | 2294 | 2448 | 3842 | 2623 | 2787 | 367 | 173 | 266 | 90 | 91 | 90 |
| 5 | -13.4 | -12.9 | -15.1 | 1973 | 2073 | 1673 | 2320 | 2659 | 2085 | 173 | 195 | 111 | 92 | 88 | 94 |
| 6 | -10.9 | -14.9 | -18.2 | 1048 | 1499 | 1891 | 1799 | 1976 | 2471 | 443 | 372 | 238 | 79 | 83 | 88 |
| 7 | -11.1 | -10.0 | -12.4 | 2499 | 1878 | 2274 | 3262 | 2623 | 2919 | 390 | 273 | 324 | 86 | 89 | 85 |
| 8 | -12.9 | -11.4 | -15.5 | 2608 | 1916 | 2584 | 3224 | 2338 | 2970 | 221 | 214 | 178 | 90 | 89 | 92 |
| 9 | -18.8 | -16.7 | -17.6 | 2793 | 1645 | 2196 | 3413 | 2027 | 2841 | 391 | 134 | 258 | 90 | 92 | 89 |
| 10 | -16.0 | -16.8 | -18.7 | 2788 | 2716 | 2865 | 3783 | 3379 | 3513 | 318 | 201 | 201 | 90 | 93 | 92 |
| Mean | -13.8 | -13.6 | -15.6 | 2229 | 1903 | 2026 | 2823 | 2321 | 2516 | 309 | 236 | 236 | 88 | 87 | 89 |
| SD | 3.1 | 3.0 | 3 | 810 | 547 | 630 | 859 | 588 | 688 | 103 | 110 | 88 | 5 | 7 | 5 |
| Median | -13.2 | -13.9 | -15.5 | 2554 | 1897 | 2196 | 3198 | 2478 | 2787 | 342 | 198 | 238 | 90 | 89 | 90 |
| IQR | -16.5 to -11.1 | -16.6 to -10.8 | -18.1 to -13.8 | 1275 to 2789 | 1423 to 2383 | 1579 to 2516 | 1876 to 3505 | 1863 to 2632 | 1931 to 2946 | 206 to 393 | 163 to 298 | 164 to 295 | 84 to 91 | 86 to 92 | 87 to 92 |

^1^ Visit cancelled due to COVID-19. GLS=global longitudinal strain, BL=baseline, D=day, M=month, GWI=global work index, GCW=global constructive work, GWW=global wasted work, GWE=global work efficiency.

**Supplemental Table S7. Patient assessments at baseline and 1-month**

| Patient | NYHA | | | KCCQ | | |
| --- | --- | --- | --- | --- | --- | --- |
|  | BL | 1M | ∆ | BL | 1M | ∆ |
| 1 | 3 | 2 | -1 | 50.0 | 55.7 | 5.7 |
| 2 | 3 | 1 | -2 | 29.2 | 80.2 | 51.0 |
| 3^(1)^ | 3 | - | - | 75.5 | - | - |
| 4 | 2 | 2 | 0 | 68.2 | 61.7 | -6.5 |
| 5 | 3 | 1 | -2 | 64.6 | 73.2 | 18.8 |
| 6 | 3 | 2 | -2 | 46.1 | 32.0 | -14.1 |
| 7 | 3 | 2 | -1 | 55.2 | 61.2 | 6.0 |
| 8 | 2 | 1 | -1 | 78.9 | 88.0 | 9.1 |
| 9 | 2 | 1 | -1 | 43.5 | 93.2 | 48.7 |
| 10 | 2 | 1 | -1 | 74.7 | 94.3 | 19.6 |
| Mean | 3 | 1 | -2 | 5.6 | 71.1 | 15.4 |
| SD | 1 | 1 | 1 | 16.4 | 20.5 | 22.3 |
| Median | 3 | 1 | -2 | 59.9 | 73.2 | 9.1 |
| IQR | 2-3 | 1-2 | - | 45.0 – 73.0 | 61.2-88 | 5.7-19.6 |

^1^ Visit cancelled due to COVID-19. BL=baseline, KCCQ=Kansas City Cardiovascular Questionnaire M=month, NYHA=New York Heart Association
