# Supplementary material for: Non-Invasive Ultrasound Therapy for Severe Aortic Stenosis: Early Effects on the Valve, Ventricle, and Cardiac Biomarkers (A Case Series)
Source: J Clin Med. 2024 Aug 7;13(16):4607. doi: 10.3390/jcm13164607 (PMC11354631; doi:10.3390/jcm13164607)
Supplement: Supplementary file 1 [file jcm-13-04607-s001.zip › 240707 Valvosoft Supplement-clean.pdf]

Non-invasive ultrasound therapy for severe aortic stenosis: early effects on the valve, ventricle, and cardiac biomarkers (a case series)

Supplemental Table S1: Inclusion and Exclusion Criteria

Inclusion Criteria	1. Subjects suffering from severe symptomatic aortic valve stenosis according to ESC 2017 definition, including subjects with a bicuspid valve.<br>2. Patient is not eligible for TAVR/SAVR according to local Heart Team.<br>3. Age ≥18 years.<br>4. Subjects who are willing to provide a written informed consent prior to participating in the study.<br>5. Subjects who can comply with the study follow up or other study requirements.
Exclusion Criteria	1. Subjects with any electrical device implanted.<br>2. Subjects with unstable arrhythmia not controlled by medical treatment.<br>3. Subjects with implanted mechanical valve in any position or bio prosthetic valve in aortic position.<br>4. Subjects with complex congenital heart disease.<br>5. Chest deformity.<br>6. Cardiogenic shock.<br>7. History of heart transplant.<br>8. Subjects requiring other cardiac surgery procedures (bypass graft surgery, mitral valve procedure, tricuspid valve procedure) within one month after treatment.<br>9. Thrombus in the heart.<br>10. Acute myocardial infarction (MI), stroke or transient ischemic attack (TIA) within one month prior to enrolment.<br>11. Subjects who are pregnant or nursing.<br>12. Subjects who are participating in another research study for which the primary endpoint has not been reached.

ESC-European Society of Cardiology, SAVR-surgical aortic valve replacement, TAVR-transcatheter aortic valve replacement

Supplemental Table S2: Procedural characteristics

Patient	Cumulative focal energy (J/mm²)	Gain IHM mean (%)	Steering mean (mm)	Peak Acoustic Power max (W)	Peak Acoustic Power mean (W)	I_{SPPA} max (W/mm²)	I_{SPPA} mean (W/mm²)	Mean Acoustic Energy Focal (J/mm²)	TZ Surface (from computation, mm²)
1	343	57	129	9120	6258	58	41	343	139
2	376	58	141	9048	7097	58	37	376	96
3	306	48	130	8543	4600	58	29	306	138
4	420	58	116	8543	4939	58	43	420	149
5	462	47	121	8469	3686	58	28	462	142
6	463	40	133	6986	3313	53	20	463	114
7	464	51	112	7552	3533	58	33	464	108
8	465	50	111	7239	3328	58	31	465	60
9	466	60	122	9120	5858	58	44	466	193
10	467	53	94	3733	2731	41	33	467	207
Mean	423	52	121	7835	4534	56	34	423	136
SD	60	6	13	1638	1467	5	7	60	44
Median	463	52	122	8506	4143	58	33	463	125
IQR	387–465	48–58	113–130	7317–8922	3379–5628	58–58	30–40	387–465	110–166

IHM= = interface human machine / therapy gain (one of the parameters that defines the level of energy of the delivered therapeutic ultrasound on the focal point), I_{SPPA}=Intensity Spatial Peak Pulse Average, TZ=target zone

Supplemental Table S3: Echocardiographic assessments at baseline, one day and one month

Patient	AVA (cm2)			MG (mmHg)			Vmax (m/sec)			AR (+)			LVEF (%)			SVi (ml/m²)			SWL (%)			Zva (mmHg/mL/m²).		
	BL	1D	1M	BL	1D	1M	BL	1D	1M	BL	1D	1M	BL	1D	1M	BL	1D	1M	BL	1D	1M	BL	1D	1M
1	0.7	0.8	0.8	33	27	28	4.0	2.3	2.5	1	1	1	45	45	49	33.5	27.2	32.3	23.1	18.3	20.3	3.5	4.4	3.3
2	0.8	0.9	0.9	33	28	31	3.7	3.4	3.6	2	1.5	1.5	45	46	57	29.9	35.9	37.9	22.3	21.8	23.7	3.95	2.8	2.7
3[1]	0.4	0.5	-	77	64	-	5.7	4.9	-	1	1.5	-	63	72	-	34.8	33.2	-	39.1	39.0	-	4.51	4.1	-
4	0.5	0.6	0.6	70	50	45	5.2	4.4	4.2	1.5	1.5	1.5	62	70	76	26.3	22.4	27.8	37.8	29.4	29.0	6.2	6.1	4.6
5	0.9	0.9	1.2	33	32	20	3.5	3.7	3.0	1.5	1.5	1.5	60	70	69	31.9	28.2	26.2	21.6	21.8	14.3	4.0	4.4	4.3
6	0.3	0.4	0.5	53	48	41	4.9	5.1	4.5	1	1	1	58	55	56	27.5	28.0	25.1	34.6	32.4	29.1	4.8	4.3	4.5
7	0.4	0.4	0.6	122	105	89	6.6	6.6	5.7	2	2	2	47	46	49	40.9	35.1	37.0	50.4	47.7	38.9	5.3	5.1	5.2
8	0.4	0.7	0.7	83	67	72	5.5	5.1	5.2	2	2	2	54	49	58	40.5	38.7	43.9	36.9	33.2	39.56	4.6	4.4	3.5
9	0.7	1.0	1.0	55	37	38	4.8	3.9	4.0	0	0	0	70	72	63	38.7	31.0	42.0	28.2	21.5	25.68	4.2	4.2	2.7
10	0.5	0.6	0.7	42	38	36	4.1	4.0	3.9	1.5	1.5	2	57	55	67	31.2	29.2	31.5	25.2	24.8	22.36	4.3	4.3	4.2
Mean	0.56	0.65	0.78	60	50	44	4.8	4.3	4.1	1.4	1.4	1.4	56	58	60	33.5	30.9	33.7	31.9	29.0	27.0	4.5	4.4	3.9
SD	0.20	0.21	0.22	29	24	22	1.0	1.2	1.0	0.59	0.55	0.61	8	12	9	5.2	4.9	6.8	9.4	9.3	8.3	0.8	0.8	0.9
Median	0.5	0.65	0.7	54	43	38	4.9	4.2	4.0	1.5	1.5	1.5	58	55	58	32.7	30.1	32.3	31.4	27.1	25.7	4.4	4.4	4.2
IQR	0.4–0.7	0.5-0.9	0.6–0.9	33–79	31-65	30–59	3.9–5.5	3.6-5.1	3.3–4.9	1–2	1–2	1–2	49–62	46-71	53–68	29.3-39.2	27.8-35.3	27.0-40.0	22.9-38.2	21.7-34.6	21.3-34.0	4.0-4.9	4.1-4.6	3.0-4.6

[1] Visit cancelled due to COVID-19. AR=aortic regurgitation, AVA=aortic valve area, D=day, LVEF=left ventricular ejection fraction, M=month, MG=mean pressure gradient, SVi=stroke volume index, SWL=stroke work loss, Zva=valvuloarterial impedance

Supplemental Table S4: Change in aortic valve parameters at one day and one month post-procedure

	AVA (cm2)		MG (mmHg)		Vmax (m/sec)		vWF Ac (%)		SVi (mL/m2)		SWL (%)		Zva (mmHg/mL/m^2)	
Patient	Δ 1D (%)	Δ 1M (%)	Δ 1D (%)	Δ 1M (%)	Δ 1D (%)	Δ 1M (%)	Δ 1D (%)	Δ 1M (%)	Δ 1D (%)	Δ 1M (%)	Δ 1D (%)	Δ 1M (%)	Δ 1D (%)	Δ 1M (%)
1	14.3	14.3	-18.8	-15.2	-42.5	-37.5	11.8	8.2	-18.8	-3.6	-20.9	-12.1	27.2	-6.4
2	12.5	12.5	-15.5	-6.1	-8.1	-2.7	26.7	15.8	20.1	26.8	-2.2	6.1	-28.6	-30.5
3[(1)]	25.0	-	-16.9	-	-14.0	-	31.6	-	-4.6	-	-0.2	-	-8.3	
4	20.0	20.0	-28.6	-35.7	-15.4	-19.2	2.9	8.7	-14.8	5.7	-22.3	-23.3	-0.7	-25.1
5	0.0	33.3	-3.0	-39.4	5.7	-14.3	53.3	22.7	-11.6	-17.9	0.9	-33.8	10.8	8.9
6	33.3	66.7	-9.4	-22.6	4.1	-8.2	17.3	2.0	1.8	-8.7	-6.4	-16.2	-10.4	-6.1
7	0.0	50.0	-13.9	-27.1	.00	-13.6	13.3	-6.1	-14.2	-9.5	-5.3	-22.9	-3.5	-1.9
8	75.0	75.0	-19.3	-13.3	-7.3	-5.5	45.8	-3.7	-4.4	8.4	-10.1	7.2	-4.1	-23.8
9	42.9	42.9	-32.7	-30.9	-18.8	-16.7	30.0	.00	-19.9	8.5	-23.7	-9.0	-0.6	-34.5
10	20.0	40.0	-9.5	-14.3	-2.4	-4.9	12.2	-2.6	-6.4	1.0	-1.2	-11.1	0.9	-3.0
Mean	24.3	39.4	-16.8	-22.7	-9.9	-13.6	24.5	5.0	-7.3	1.2	-9.13	-12.8	-1.7	-13.6
SD	22.3	22.1	8.9	11.3	14.1	10.6	16.1	9.7	11.8	13.1	9.65	13.4	14.3	15.1
Median	20.0	40.0	-16.17	-22.6	-7.69	-13.63	22.00	2.00	-9.0	1.0	-5.9	-12.1	-2.1	-6.4
IQR	9.4–35.7	17.1–58.3	-21.6– -9.50	-33.3– -13.8	-16.2–1.0	-18.0–5.2	12.1–35.1	-3.2 12.3	-15.8– -2.9	-9.1– 8.5	-21.2– -1.0)	-23.1 – -1.4	-8.9–3.4	-27.8– -2.4

[1] Visit cancelled due to COVID-19. Delta (Δ) is the percent change of parameters at one day (1D) or at one month (1M) compared to baseline. Negative Δ represent at decrease. AVA=aortic valve area, D=day, M=month, MG=mean pressure gradient, SVi=stroke volume index, SWL=stroke work loss, vWF Ac=von Willebrand factor activity, Zva=valvuloarterial impedance

Supplemental Table S5. Laboratory assessments at baseline, one day, and one month

Patient	BNP (pg/ml)			Troponin-T (µg/L))			vWF activity (%)			CRP (mg/L)		
	BL	1D	1M	BL	1D	1M	BL	1D	1M	BL	1D	1M
1	223	174	251	26	31	24	110	123	119	1.2	0.9	1.4
2	258	246	158	23	20	22	120	152	139	4	5.9	2.3
3(1)	521	279	-	20	40	-	114	150		1.7	1.4	
4	313	428	328	27	124	32	138	142	150	1.1	5	1.6
5	114	133	115	20	31	24	150	230	184	4.1	5.2	3.4
6	854	470	507	54	56	56	150	176	153	1.9	10	3
7	1414	-	1043	14	46	34	98	111	92	0.7	9.2	0.9
8	1000	793	1058	32	28	32	107	156	103	2.8	7.6	4.5
9	72	102	42	30	34	28	150	195	150	1.7	1.4	1.8
10	1032	491	239	29	60	27	115	129	112	5.1	4.8	1.2
Mean	580	346	416	28	47	31	125	156	134	2.4	5.1	2.2
SD	463	221	384	111	30	10	20	36	29	1.5	3.2	1.2
Median	417	279	251	27	37	28	118	151	139	1.8	5.1	1.8
IQR	196-10080	154-481	137-775	20-31	30-57	24-33	109-1500	128-181	108-152	1.2-4.0	1.4-8.0	1.3-3.2

[1] Visit cancelled due to COVID-19. BNP=brain natriuretic peptide, CRP=C-reactive protein, D=day, M=month, vWF=von Willebrand factor

Supplemental Table S6. Left ventricular myocardial mechanic assessed by two-dimensional speckle-tracking at baseline, one day and one month

Patient	GLS (%)			GWI (mmHg%)			GCW (mmHg%)			GWW (mmHg%)			GWE (%)		
	**BL**	**1D**	**1M**	**BL**	**1D**	**1M**	**BL**	**1D**	**1M**	**BL**	**1D**	**1M**	**BL**	**1D**	**1M**
1	-10.2	-9.4	-10.0	1321	1196	816	1902	1526	1284	400	481	396	81	70	78
2	-11.2	-11.0	-15.4	1139	1158	1485	1513	1441	1776	216	133	150	85	87	91
3[(1)]	-15.7	-16.6	-	2737	2652	-	3171	2618	-	175	188	-	94	92	-
4	-18.0	-16.0	-18.0	3381	2294	2448	3842	2623	2787	367	173	266	90	91	90
5	-13.4	-12.9	-15.1	1973	2073	1673	2320	2659	2085	173	195	111	92	88	94
6	-10.9	-14.9	-18.2	1048	1499	1891	1799	1976	2471	443	372	238	79	83	88
7	-11.1	-10.0	-12.4	2499	1878	2274	3262	2623	2919	390	273	324	86	89	85
8	-12.9	-11.4	-15.5	2608	1916	2584	3224	2338	2970	221	214	178	90	89	92
9	-18.8	-16.7	-17.6	2793	1645	2196	3413	2027	2841	391	134	258	90	92	89
10	-16.0	-16.8	-18.7	2788	2716	2865	3783	3379	3513	318	201	201	90	93	92
Mean	-13.8	-13.6	-15.6	2229	1903	2026	2823	2321	2516	309	236	236	88	87	89
SD	3.1	3.0	3	810	547	630	859	588	688	103	110	88	5	7	5
Median	-13.2	-13.9	-15.5	2554	1897	2196	3198	2478	2787	342	198	238	90	89	90
IQR	-16.5 to -11.1	-16.6 to -10.8	-18.1 to -13.8	1275 to 2789	1423 to 2383	1579 to 2516	1876 to 3505	1863 to 2632	1931 to 2946	206 to 393	163 to 298	164 to 295	84 to 91	86 to 92	87 to 92

[1] Visit cancelled due to COVID-19. GLS=global longitudinal strain, BL=baseline, D=day, M=month, GWI=global work index, GCW=global constructive work, GWW=global wasted work, GWE=global work efficiency.

Supplemental Table S7. Patient assessments at baseline and 1-month

Patient	NYHA			KCCQ		
	BL	1M	Δ	BL	1M	Δ
1	3	2	-1	50.0	55.7	5.7
2	3	1	-2	29.2	80.2	51.0
3[(1)]	3	-	-	75.5	-	-
4	2	2	0	68.2	61.7	-6.5
5	3	1	-2	64.6	73.2	18.8
6	3	2	-2	46.1	32.0	-14.1
7	3	2	-1	55.2	61.2	6.0
8	2	1	-1	78.9	88.0	9.1
9	2	1	-1	43.5	93.2	48.7
10	2	1	-1	74.7	94.3	19.6
Mean	3	1	-2	5.6	71.1	15.4
SD	1	1	1	16.4	20.5	22.3
Median	3	1	-2	59.9	73.2	9.1
IQR	2-3	1-2	-	45.0 – 73.0	61.2-88	5.7-19.6

[1] Visit cancelled due to COVID-19. BL=baseline, KCCQ=Kansas City Cardiovascular Questionnaire M=month, NYHA=New York Heart Association
