# Supplementary figures and images for: Non-Invasive Ultrasound Therapy for Severe Aortic Stenosis: Early Effects on the Valve, Ventricle, and Cardiac Biomarkers (A Case Series)
Source: J Clin Med. 2024 Aug 7;13(16):4607. doi: 10.3390/jcm13164607 (PMC11354631; doi:10.3390/jcm13164607)

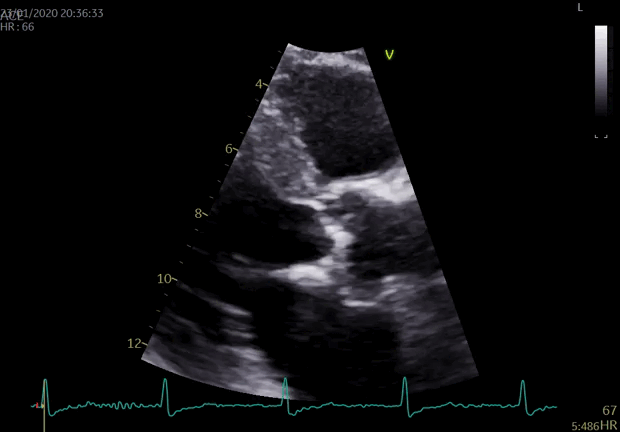

Supplement: Supplementary file 1 [file jcm-13-04607-s001.zip › Supplemental Movie 3 MS baseline.gif]

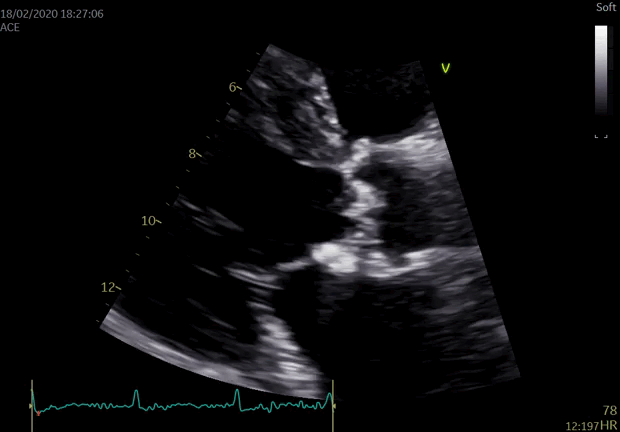

Supplement: Supplementary file 1 [file jcm-13-04607-s001.zip › Supplemental movie 4 MS post procedural.gif]
